# Whole-genome sequencing and ad hoc shared genome analysis of *Staphylococcus aureus* isolates from a New Zealand primary school

**DOI:** 10.1038/s41598-021-99080-8

**Published:** 2021-10-13

**Authors:** Pippa Scott, Ji Zhang, Trevor Anderson, Patricia C. Priest, Stephen Chambers, Helen Smith, David R. Murdoch, Nigel French, Patrick J. Biggs

**Affiliations:** 1 Department of Pathology and Biomedical Science, University of Otago, Christchurch, New Zealand; 2 School of Veterinary Science, Massey University, Palmerston North, New Zealand; 3 Canterbury Health Laboratories, Canterbury District Health Board, Christchurch, New Zealand; 4 Department of Preventive and Social Medicine, University of Otago, Dunedin, New Zealand; 5 School of Fundamental Sciences, Massey University, Palmerston North, New Zealand

**Keywords:** Computational biology and bioinformatics, Infectious diseases

## Abstract

Epidemiological studies of communicable diseases increasingly use large whole-genome sequencing (WGS) datasets to explore the transmission of pathogens. It is important to obtain an initial overview of datasets and identify closely related isolates, but this can be challenging with large numbers of isolates and imperfect sequencing. We used an ad hoc whole-genome multi locus sequence typing method to summarise data from a longitudinal study of *Staphylococcus aureus* in a primary school in New Zealand. Each pair of isolates was compared and the number of genes where alleles differed between isolates was tallied to produce a matrix of “allelic differences”. We plotted histograms of the number of allelic differences between isolates for: all isolate pairs; pairs of isolates from different individuals; and pairs of isolates from the same individual. 340 sequenced isolates were included, and the ad hoc shared genome contained 445 genes. There were between 0 and 420 allelic differences between isolate pairs and the majority of pairs had more than 260 allelic differences. We found many genetically closely related *S. aureus* isolates from single individuals and a smaller number of closely-related isolates from separate individuals. Multiple *S. aureus* isolates from the same individual were usually very closely related or identical over the ad hoc shared genome. Siblings carried genetically similar, but not identical isolates. An ad hoc shared genome approach to WGS analysis can accommodate imperfect sequencing of the included isolates, and can provide insights into relationships between isolates in epidemiological studies with large WGS datasets containing diverse isolates.

## Introduction

Epidemiological studies that investigate the transmission and evolution of pathogenic bacteria increasingly include the use of bacterial whole genome sequencing (WGS), and can generate huge amounts of data. This is particularly true outside of outbreak investigations when investigations are focused on asymptomatic bacterial carriage. We previously published descriptive data for a cohort study about the carriage of *Staphylococcus aureus* in a primary school in New Zealand^1^. Subsequently, *S. aureus* isolates obtained in the study have been sequenced, producing WGS data for over 300 isolates. In data such as these, there are likely to be both closely related isolates (as individuals are swabbed repeatedly over the study) as well as isolates that differ greatly (as in the absence of a disease outbreak there is likely to be considerable diversity in isolates carried by individuals in the population). The first step in the analysis of such a dataset would be a descriptive analysis that gives an overview of similarities and differences across the full set of isolates. Ideally such an analysis would include as many of the isolates as possible, quantify the extent of differences between each isolate pair and allow for the identification of clusters of closely related isolates while still capturing isolates that are unrelated to others.

Summarising WGS data concisely from (non-outbreak) epidemiological studies in a way that satisfies these criteria is challenging. Comparisons of isolates based on accessory genomes, single nucleotide polymorphisms (SNPs), recombinations and deletions in such large datasets lack the means to quantify differences between isolates in a way that can be applied across the whole dataset. In contrast, conventional methods such as 7-locus multi locus sequence typing (MLST), which look at a small number of core bacterial genes, provide a broad overview of a large number of isolates but provide coarse resolution only, grouping together isolates that may not be closely related^2–4^. Better resolution could be achieved by using a larger number of pre-defined loci in the MLST scheme. However, a prerequisite for this approach would be that the pre-defined genes exist in every isolate that is to be included. Isolates where there is uncertainty about the nucleotide sequences in any one of the pre-defined loci would be completely excluded from such an analysis. “Ad hoc shared genome” approaches have the potential to overcome the exclusion of large numbers of sequenced isolates, such as in analyses using pre-defined loci, by comparing only genes that are both present and have a high certainty over nucleotide sequences in all isolates (as genes might be falsely absent due to incorrect assembly or incomplete coverage of the gene)^5^. Such ad hoc approaches also provide better resolution than 7-locus MLST^6,7^. We used an ad hoc shared genome method to compare *S. aureus* isolates obtained from a longitudinal study in a primary school, in order to obtain an overview of the dataset and identify closely related isolates.

## Methods

### Study participants

The study was carried out in a primary school in Christchurch, New Zealand^1^. Children in four classes of 8–10 year olds were invited to participate in the study. Teachers and teacher aides in these classes were invited to participate, as well as members of the research team who assisted with swabbing. Informed consent was obtained from adult participants and from parents/guardians of participating children. The study was approved by the New Zealand Northern A Health and Disability Ethics Committee (14/NTA/218) and all methods were performed in accordance with relevant national and international guidelines and regulations for the inclusion of human participants in research, including the Declaration of Helsinki.

### Microbiological sampling

**Figure 1:**
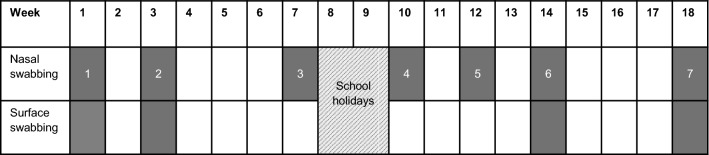
Sample collection schedule.

Nasal swabs were taken from participants on 7 occasions between February 16 2015, and June 17 2015. There were 2–4 weeks between each sampling round (Fig. 1). Swabs were taken from the anterior nares using a sterile, gel-moistened swab (COPAN Transystem, Amies transport media). Both nostrils were sampled using the same swab. If the participant reported a skin lesion with exudate that was in an area that would not be covered by shorts and a T-shirt, the lesion was swabbed using a sterile gel-moistened swab. At rounds 1, 2, 6 and 7, samples were taken from surfaces in the classrooms. We tested surfaces that we observed to have frequent contact with hands (particularly those of children). This included handles of doors, drawers, and taps/faucets, couch arms, table tops, laptop keyboards, musical instruments, and carpets where children sit during group activities. Each of these surface samples was taken by rolling a gel-moistened swab over the surface for approximately 10 s. Swabs were stored and transported at room temperature for laboratory processing within 3 h of sampling.

### Microbiological testing

Swabs were processed at Canterbury Health Laboratories, Christchurch. Samples were enriched by incubation at 36 °C in a brain heart infusion (BHI) broth containing mannitol, colistin and aztreonam and, after a minimum of 16 h, streaked onto CHROMagar *S. aureus* media (Fort Richard, Auckland, New Zealand). The next day, plates were checked for the growth of pink colonies indicative of *S. aureus*. The identity of *S. aureus* was confirmed using the coagulase test and, occasionally, by MALDI-TOF (Matrix Assisted Laser Desorption/Ionization-Time of Flight, Bruker) testing.

A sample of each visually distinct *S. aureus* isolate was loaded in to the Protect microorganism preservation system (ThermoFisher Scientific) and stored at − 80 °C. A sample of each enrichment broth from which *S. aureus* was obtained was also stored at − 80 °C. A group of 25 isolates from round 3 and their associated broths were discarded in error. For these samples, information about the detection of *S. aureus* and the antimicrobial resistance (from disc diffusion) of the isolates are available, but no genetic data could be produced.

### Whole genome sequencing

Each stored *S. aureus* isolate was cultured and pre-treated with Lysostaphin (Sigma-Aldrich) before DNA was extracted from the GenElute Bacterial Genomic DNA Kit (Sigma-Aldrich) as described in the manufacturer’s protocol. DNA quality was checked using gel diffusion (to confirm a single band of large molecular weight DNA could be observed) and NanoDrop 1000 (Thermo Fisher Scientific) spectrophotometry.

In addition to the stored isolates, sub-culture isolates were obtained from nine of the stored enrichment broth samples in order to investigate diversity of *S. aureus* isolates from a single swab. Single broth samples were selected from three individuals that were carrying *S. aureus* with the same antibiotic resistance pattern at every round, from one that had a changing antibiotic resistance pattern over the study and from one that appeared to acquire *S. aureus* nasal carriage during the study. Two broth samples were selected from two individuals who were potentially simultaneously carrying two strains of *S. aureus*: both had two visually distinct colony types from a single swab, and one had a different antibiotic resistance pattern from these colonies. Each of the selected broths were plated on blood agar and six *S. aureus* colonies selected for DNA extraction. The colonies were obtained from different parts of the plate, selecting a range of visual appearances. DNA from these sub-cultures was then extracted and isolates stored as described above.

Illumina Nextera XT libraries were prepared at New Zealand Genomics Limited (NZGL), Massey University, Palmerston North, New Zealand. Quality checks for library sample pooling were performed using an Illumina MiSeq, and sequencing with an Illumina HiSeq (2 × 125 base) v4 at NZGL, University of Otago, Dunedin, New Zealand.

We had a small number of spare spaces available in our final sequencing run. To fill these spaces, we identified isolates we considered to be of high-importance (e.g. a single isolate obtained from an individual) but had in earlier sequencing runs produced more contigs than the majority of the isolates. We then re-extracted and re-sequenced these isolates.

### De novo assembly, genome annotation, MLST and ad hoc whole-genome MLST (wgMLST) analysis

**Figure 2:**
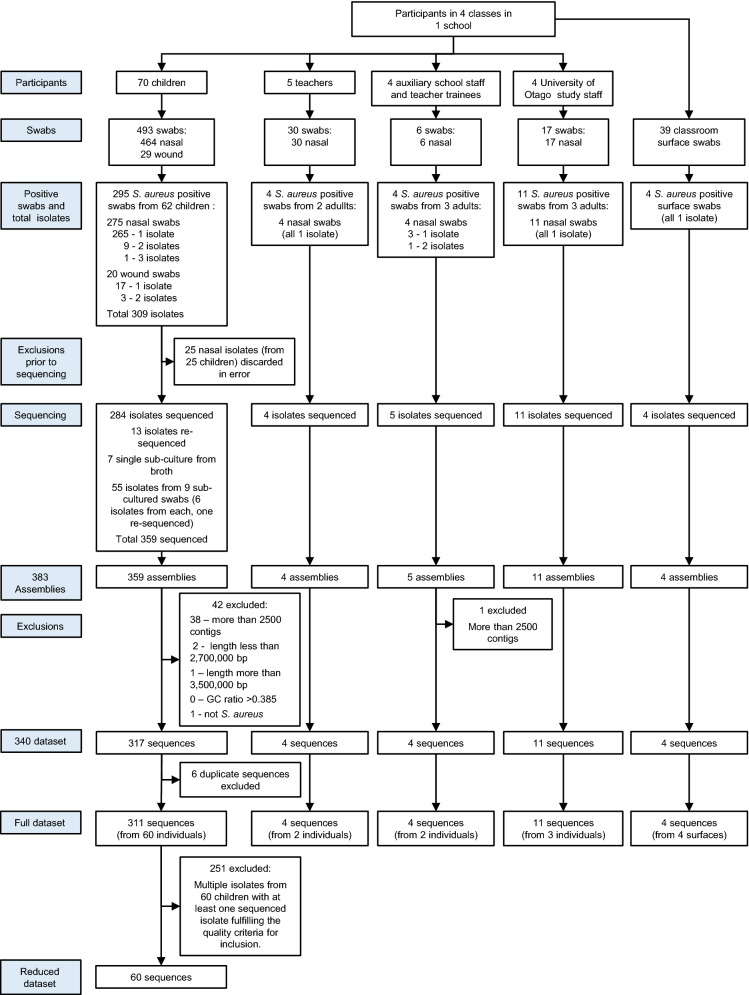
Isolate flow chart.

The sequencing raw reads were quality-control processed and quality evaluated with QCtool^8^. The processed reads were assembled de novo with SPAdes (version: 3.9.0)^9^ in “careful mode” to correct mismatches. We retained samples where there were 2500 or fewer contigs, the total length was between 2700 and 3500 kb, and the GC ratio less than 37.5% (Fig. 2). These limits were chosen pragmatically to include 90% (contigs and total length) or 95% (GC ratio) of the sequenced isolates in order to exclude as few isolates as possible from the analysis.

The assemblies were annotated with Prokka (version 1.11)^10^. The MLST Sequence Types (ST) of the isolates were determined using the online tool “Sequence query” on the PubMLST website^11^. The complete chromosome sequence of *S. aureus* subsp. aureus MW2 (GenBank Acc. NC_003923) was used as the reference genome and genomic analyses were performed with fast-GeP, a rapid, efficient ad hoc wgMLST method^12,13^. Average depth of coverage and the percentage of reference mapped were produced for each isolate using Samtools (version 1.13)^14^, and the same reference genome.

For each sequenced isolate, the presence or absence of each of 2623 genes in the reference genome was assessed and, where the gene was present, the allele identified and recorded. Genes that were present in all of the included sequenced isolates were considered to be part of the ad hoc shared genome. Isolates were compared in a pair-wise fashion and the number of genes where alleles differed between each pair was tallied to produce a matrix of “allelic differences”.

### Analysis

We excluded data that represented duplicate sequencing runs on the same isolate (Fig. 2), retaining the sequence for which there were the fewest contigs produced for the isolate in WGS. We plotted histograms of the number of allelic differences between isolates for: the full dataset; pairs of isolates of different conventional 7-locus ST profiles; pairs of isolates of different ST profiles; pairs of isolates from different individuals; and pairs of isolates from the same individual.

We constructed equal angle phylogenetic network plots using SplitsTree4 (version 4.14.6)^15,16^. The genetic distance was calculated for each pair of isolates using the Jaccard dissimilarity index^17^, and is interpreted as the percentage of unshared alleles. We used a permutation test to assess whether isolates from siblings have smaller genetic distances to random pairings of isolates in these data. In order to avoid over-representation of individuals with several isolates, we used a reduced dataset for this analysis. This dataset contained one randomly selected isolate from each child for whom we had at least one sequenced isolate that fulfilled the quality criteria for inclusion. These statistical analyses were performed using R Statistical Software (version 4.0.0)^18^.

## Results

### Participants and samples

A total of 70 children and 13 adults were enrolled in the study (Fig. 2). Children contributed 493 swabs (464 nasal and 29 skin lesion swabs) to the study and adults 53 (all nasal swabs). Fifty-eight (83%) participating children contributed at all rounds of nasal swabbing; 7 children were absent from school on a single swabbing day, 2 were absent twice, and 3 children left the school after contributing swabs in rounds 1 and 2 only. Additionally, there were 29 skin lesion swabs taken which were all from children. Of the 5 teachers, 2 contributed at all swabbing rounds, 2 were absent from a single swabbing round, and 1 contributed only from round 4 onwards. The 4 auxiliary staff (teacher aides and student teachers) only contributed once or twice each and the 4 members of the study team contributed either at all rounds (2 staff) or only once or twice, reflecting the number of sampling rounds they assisted in swab collection at the school. There were 39 environmental swabs taken. Details about the basic epidemiology of carriage in this study are reported elsewhere^1^.

### Dataset construction

Of the swabs, 318 were positive for *S. aureus* (Fig. 2). Thirteen of these had 2 morphologically distinct isolates obtained (9 child and 1 adult nasal swabs, 3 child wound swabs) and 1 had 3 morphologically distinct isolates (1 child nasal swab). DNA was extracted and sequenced for 383 isolates (Fig. 2) and 340 sequenced isolates fulfilled the quality criteria for inclusion (90% of sequenced isolates had fewer than 1000 contigs, 82% had a total length between 2700 and 3300 kb, and 90% had a GC ratio of 32.5–34.0%). Quality measures for the 383 isolates are summarised in Supplementary Table S1, and the full matrix of allelic differences is provided in Supplementary Table S2. The ad hoc shared genome contained 445 genes. A further 6 isolates that were sequenced twice had one sequence excluded (retaining the sequence with the fewest contigs) to form the full dataset at this point. Of the 334 isolates included in the full dataset, 213 (64%) had fewer than 500 contigs and are referred to in this report as “higher quality sequences”, and 121 (36%) had more than 500 contigs and are referred to as “lower quality sequences”. Higher quality sequences had a median average-depth-of-coverage over all positions of 93 (IQR 74–107), lower quality sequences 70 (IQR 48–88), and those excluded from the full data set 30 (IQR 10–86). The percentage of the reference genome mapped was similar in each of these groups with a median of 90% (IQR 86–91) in each of the 3 groups.

### Differences between isolate pairs

**Figure 3:**
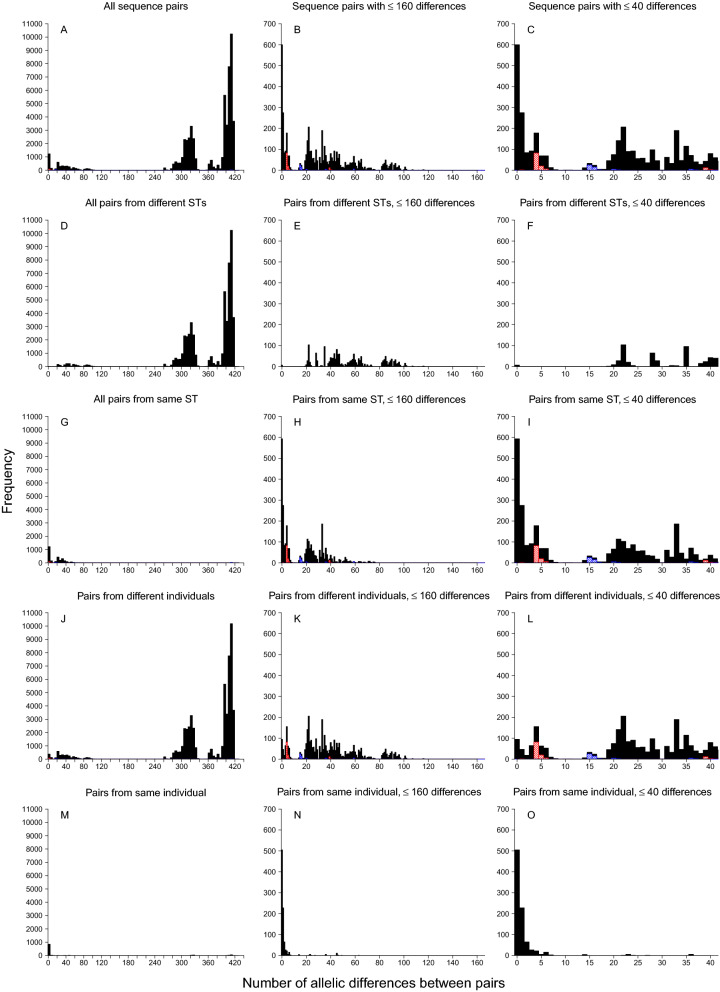
Histograms of allelic differences between sequence pairs. Red hashed bars indicate comparisons of isolates from siblings. Blue hashed bars indicate comparisons of isolates from a child participant and a study staff member (unlikely to represent direct transmission).

There were between 0 and 420 allelic differences (out of 445 shared genes) between each pair of isolates (Fig. 3, panel A). The majority of isolate pairs had more than 260 allelic differences. There was a complete absence of isolate pairs with between 100 and 260 allelic differences. There were very few isolate pairs with between 7 and 15 allelic differences (Fig. 3, Panel A–C), and most of the pairs in this range included at least one lower quality sequence (Supplementary Figure 1, panels F and I).

### Differences between isolates of the same MLST profile

**Figure 4:**
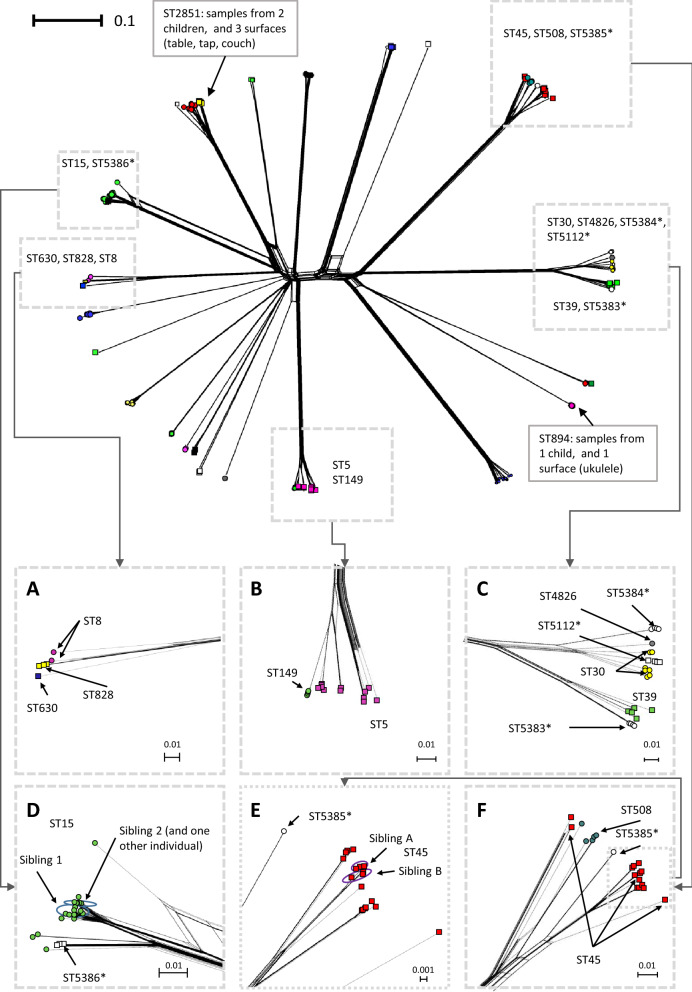
Phylogenic network plot, full dataset. Isolates from the same ST have same shape and colour. Symbols with no fill and/or ST number with asterisk indicate MLST types newly registered from this study.

A total of 36 conventional 7-locus MLST profiles were identified in this population (Table 1). The isolate pairs with the highest numbers of allelic differences were from different MLST profiles (Fig. 3, panels D–F). However, there was overlap of the number of allelic differences between pairs from different MLST profiles and pairs from the same MLST profiles (Fig. 3, panels D–I). This overlap occurred between 20 and 80 allelic differences (Fig. 3, panels E and H) and pairs from different MLST profiles with the fewest differences tended to be from MLSTs from the same clonal complex (Table 2). However, there were some exceptions to this, with some pairs from MLSTs within the same clonal complex having many allelic differences (e.g. ST5 and ST6). The ambiguity between extent of relatedness and designated MLST profile is also illustrated in the phylogenetic network plot, Fig. 4 (inset A, B, C and F). The MLSTs within which there were the most allelic differences between isolates were ST45 (Table 2 and Fig. 4, inset F), and ST8 (Table 2 and Fig. 4, inset A). The higher resolution wgMLST method also shows that there are more wgMLST allelic differences within the 7-locus ST45 than between isolates from ST45 and other 7-locus MLSTs from the same clonal complex (Table 2 and Fig. 4, inset F).

**Table 1:** Frequency of conventional 7-locus MLST profiles in the 340 included isolates.

7-locus MLST type	Number of isolates
15	38
59	32
45	30
5	28
6	20
1	19
78	17
30	13
25	10
97	10
894	9
2851	9
12	8
39	8
188	8
508	8
22	7
149	8
4826	6
5112*	6
779	5
5383*	5
5384*	5
5386*	5
828	4
5111*	4
72	3
5385*	3
8	2
121	2
672	2
5388*	1
630	1
2276	1
5387*	1
5390*	1

*MLST type newly registered from this study.

**Table 2:** Observed allelic differences between MLSTs in the same or different clonal complexes.

Clonal complexes present in dataset^19^	7-Locus MLSTs present in dataset (n wgMLST allelic differences within MLST)		7-Locus MLST pairs with 15–120 allelic differences in wgMLST (n wgMLST allelic differences, n 7-locus MLST loci differences)		7-Locus MLST pairs with 280–420 allelic differences in wgMLST (n wgMLST allelic differences, n 7-locus MLST loci differences)	
CC1	1	(0–42)	1:2851	(39–62, 1)	1:188	(260–267, 2)
	188	(0–38)	1:5388	(45–65, 1)	188: 2851	(262–265, 3)
	2851	(0–4)	2851:5388	(82–84, 2)	188: 5388	(273–275, 2)
	5388	(n/a)				
CC5	5	(0–61)	5: 149	(28–59, 1)	5:6	(322–333, 2)
	6	(0–29)			6:149	(323–325, 3)
	149	(0)				
CC8	8	(79)	8:630	(112–115, 1)	8:72	(323–383, 3)
	72	(1–48)	8:828	(22–89, 1)	72:630	(319–323, 3)
	630	(n/a)	630:828	(116–117, 2)	72:828	(326–332, 4)
	828	(0–3)				
CC15	15	(0–58)	15:5386	(19–54, 1)		–
	5386	(0–1)				
CC22	22	(0–2)	22:5387	(45–46, 1)		–
	5387	(n/a)				
CC30	30	(0–45)	30:39	(85–97, 2)		–
	39	(0–48)	30:4826	(43–40, 1)		
	4826	(0)	30:5112	(38–43, 1)		
	5112	(0–2)	30:5383	(92–94, 3)		
	5383	(0)	30:5384	(54–60, 2)		
	5384	(1–2)	39:4826	(83–95, 3)		
			39:5112	(82–94, 3)		
			39:5383	(44–53, 1)		
			39:5384	(95–108, 4)		
			4826:5112	(42–43, 2)		
			4826:5383	(90, 4)		
			4826:5384	(58–59, 2)		
			5112:5383	(88–89, 4)		
			5112:5384	(56–58,3)		
			5383:5384	(101–102, 4)		
CC45	45	(0–76)		(56–74, 1)		–
	508	(0–53)		(44–73, 1)		
	5385	(0)		(60–70, 1)		
CC97	97	(0–38)		–		–
Unclassified	12	(0–1)		(97–98, 2)	All remaining 7-locus MLST pairs (including between clonal complexes)	
	25	(0–3)				
	59	(0–41)				
	78	(0–28)				
	121	(1)				
	672	(2)				
	779	(0–1)				
	894	(0–3)				
	2276	(n/a)				
	5111	(0–3)				
	5390	(n/a)

### Differences between isolates from the same individual

The vast majority of isolate pairs from the same individual (53 individuals with isolate pairs, both from different time points and from multiple sub-cultures from the same swab) had fewer than 5 allelic differences in the 445 genes compared (Fig. 3, panels M–O). From subcultures from the same swab, most isolates had only 0 or 1 allelic difference. A similar pattern was seen across swabs over from the same individual (taken at different sampling rounds), except for a slightly larger proportion having 2 or 3 allelic differences (data not shown). The few isolate pairs from the same individual that displayed many allelic differences (Fig. 3, panel M) reflect the carriage of multiple unrelated MLST profiles by the individual either simultaneously or in sequence over time. Higher quality isolate pairs from the same individual had 0–4 differences or more than 300 differences, while lower quality pairs had 0–7 differences (with the majority 0 or 1 differences), between 20 and 70 differences, or more than 290 differences (Supplementary Figure 2, panels D–I).

### Differences between isolates from different individuals

The vast majority of isolate pairs from different individuals had more than 250 allelic differences (Fig. 3, Panel J). In the region of the histogram showing few allelic differences there were peaks in frequency (Fig. 3, panels J–L): one peak at around 0 to 7 differences (with 25 individuals and 4 environmental swabs contributing to at least one isolate pair), which is similar to the number of allelic differences seen between isolates from the same individual (Fig. 3, panel M–O); and several other peaks between 14 and 100 differences. Within the histogram peak at 0 to 7 allelic differences, were a substantial number of isolate pairs from the 2 sets of siblings in the study population (each sibling set had 2 members). The majority of comparisons between isolates from siblings showed 3 to 6 differences with a small number between 35 and 40 different (marked in red, Fig. 3, panel L). There were no comparisons that had fewer than 3 allelic differences between isolates from siblings. Isolates from sibling pairs were more genetically similar than isolates from other pairs of individuals (permutation test *p* ≤ 0.0001).

Among individuals from whom *S. aureus* was repeatedly isolated, there were 2 groups of individuals with very similar isolates (8 or fewer allelic differences). These groups consisted of a pair (siblings) and a group of 4 (2 of whom were siblings). The next most closely related isolates from repeat carriers had 15–18 allelic differences (2 pairs of repeat carriers, with one of the pairs including a research team member, (Figs. 3, 4), who had had no previous contacts to the school or local community meaning it was unlikely to be the result of direct transmission. One repeated carrier was associated with 3 of the surface swab isolates.

### Single isolates from a source

There were 51 occurrences of a 7 locus MLST type being isolated only once from a particular participant or surface (42 from children, 5 from adults and 4 from different classroom surfaces). Of these, 25 had more than 18 allelic differences from all other isolates in the dataset (17 of which were of very good quality with fewer than 500 contigs). The remaining 26 isolates were more similar to isolates from one or more other study participants and had 18 or fewer differences to isolates from the other participants.

## Discussion

Using an ad hoc shared genome approach we were able to obtain an overview of *S. aureus* WGS data obtained in a longitudinal study in a New Zealand primary school. We found many closely genetically related *S. aureus* isolates from single individuals and a smaller number of closely related isolates collected from separate individuals. Multiple *S. aureus* isolates from the same individual, both from the same swab as well as over the duration of the study, tended to be very closely related or identical over the ad hoc shared genome. Siblings carried genetically similar, but not identical, isolates as did some individuals who did not live with each other.

The major strength of this analysis is that we were able to include the vast majority (89%) of our 383 sequenced isolates. Conversely, including isolate sequences of variable quality reduced the number of genes (and consequently, the proportion of the whole genome) in the ad hoc shared genome. It could be argued that the resolution of our analysis could be improved if higher quality of genome assemblies were used (e.g. by using a lower number of contigs as the cut-off for inclusion in the analysis). However, in a study where a large number of genome are sequenced it is likely that some isolates will be imperfectly sequenced and our results have shown that Fast-GeP could accommodate lower quality genome assemblies while maximising the number of included isolates from the maximum number of study participants. Although the exact number of allelic differences between isolates might be challenging to interpret when one or both isolates were imperfectly sequenced, our analysis shows that when few or very few (3–8 and 0–2 differences in this analysis respectively) differences are observed between isolates, the isolates are closely related enough to be considered, for example, part of the same transmission chain in a transmission study, even if quality of sequencing is of lower quality. This means the inclusion of these isolates can still provide meaningful epidemiological data without resequencing being essential. Only the very small number of imperfectly sequenced pairs (177 pairs from a total of 334 isolates and 55,611 pairs) with somewhat higher numbers of differences (e.g. 15–40 in this analysis) cannot be ruled in or out as being part of a transmission chain and therefore do not add interpretable information to a study of transmission. In contrast, excluding all imperfectly sequenced isolates would result in only 213 isolates and 25,578 isolate pairs being available for analysis.

Another advantage of this analysis is that it allowed us to easily compare isolates and rapidly identify clusters of very similar isolates even within the same 7-locus MLST profile. This meant we were able to quickly identify pairs or groups of samples that might represent transmissions, which can be earmarked for more in-depth genetic analysis if required. It also provided us with a higher resolution view of the relatedness between conventional 7-locus MLST types, showing in places more or fewer similarities than would be expected from looking at the 7 loci and clonal complex information. Although higher resolution is to be expected with WGS methods compared to 7-locus MLST, this dataset shows that it is possible to obtain a high resolution view of relatedness between isolates even with imperfect sequencing. As an allele-based method, wgMLST has many advantages such as fast, high efficiency, portability, scalability, high resolution and unambiguous nature^2–4,20^. Moreover, it could to some extent accommodate the noise caused by recombination as multiple genetic differences in the recombinant allele sequence would collapse into a single allele difference^3,21^. Fast-GeP itself has a distinct speed advantage to the older generation of ad hoc wgMLST programs, such as Genome Profiler^22^, requiring much less time to analyse the same genome sequences^13^.

The downside of the ad hoc shared genome (or other allele based) approach is that sequence variations in different alleles of each gene are not examined. However, the approach of summing allelic differences provides a broad summary of the relatedness of isolates and this information can then be used to identify groups of isolates to be looked at in more detail if required.

The ad hoc shared genome method has allowed us to assess patterns of *S. aureus* carriage longitudinally in a large, interacting cohort of healthy individuals in a primary school setting. We observed distinct peaks in the frequency of allelic differences, with groups characterised by:

1. Very few differences (0–2 allelic differences: mainly isolates from the same individual),
2. Few differences (3–8 allelic differences: isolates from different individuals including siblings, and isolates with lower quality sequencing from the same individual),
3. Frequent differences (15–120 allelic differences: isolates for the same 7-locus ST profile, a few from different ST profiles, and a very small number from isolates with lower quality sequencing), and
4. Many differences (280–420 allelic difference: isolates from different MLST profiles).

These patterns in isolates compared in each peak region suggest that the number of allelic differences can help to identify isolates or groups for further investigation. For example, our data suggests that while 3–8 allelic differences may indicate transmission between individuals, 2 or fewer allelic differences between isolates from different sources may be associated with non-established transmissions, or an individual or a surface effectively acting as a fomite with no further detections of related isolates from the same source in subsequent swabs. This is further supported by the substantial numbers of genotypes that were isolated only once and only from a single source, suggesting that *S. aureus* was temporarily acquired from a source that was not one of our participants. However, further research is needed to determine if other epidemiological studies have similar findings. It is important to note that in different studies, which will have different numbers of genes in the ad hoc shared genome, peaks would coincide with different numbers of allelic differences.

Allelic profiling methods similar to the fast-GeP method have been used to examine bacterial WGS data to investigate outbreaks of bacterial disease^5,23^, as have SNP-based methods^24^. However, to our knowledge, this is the first (longitudinal) cohort study in healthy, community-based interacting population, where WGS is used to examine patterns of relatedness of an asymptomatic carried bacterial pathogen. Despite differences in methods, our findings are similar where there are comparable aspects in other studies. For example, we found subcultures from the same swab generally had fewer differences than isolates from swabs from each individual over time, which is compatible with findings of increasing diversity of the within-host methicillin-resistant population with long term carriage^24^.

## Conclusion

An ad hoc shared genome approach to WGS analysis can accommodate imperfect sequencing of the included isolates, and can provide insights into patterns of relationships between isolates in epidemiological studies with large WGS datasets containing diverse populations of isolates.

## Supplementary Information

Supplementary Figures.

Supplementary Table S1.

Supplementary Table S2.
