# Supplementary material for: Whole-genome sequencing and ad hoc shared genome analysis of Staphylococcus aureus isolates from a New Zealand primary school
Source: Sci Rep. 2021 Oct 13;11:20328. doi: 10.1038/s41598-021-99080-8 (PMC8514452; doi:10.1038/s41598-021-99080-8)
Supplement: Supplementary file 1 — Supplementary Figures. [file 41598_2021_99080_MOESM1_ESM.pdf]

# Supplementary figures

# List of Figures

1 Histograms of allelic differences between sequence pairs, stratified by sequencing quality . . . . . . . . . . . . . . . . . . . . . . . . . . . . . . 2

2 Histograms of allelic differences between sequence pairs from the same individual, stratified by sequencing quality . . . . . . . . . . . . . . . . . . 3

# Supplementary figure 1

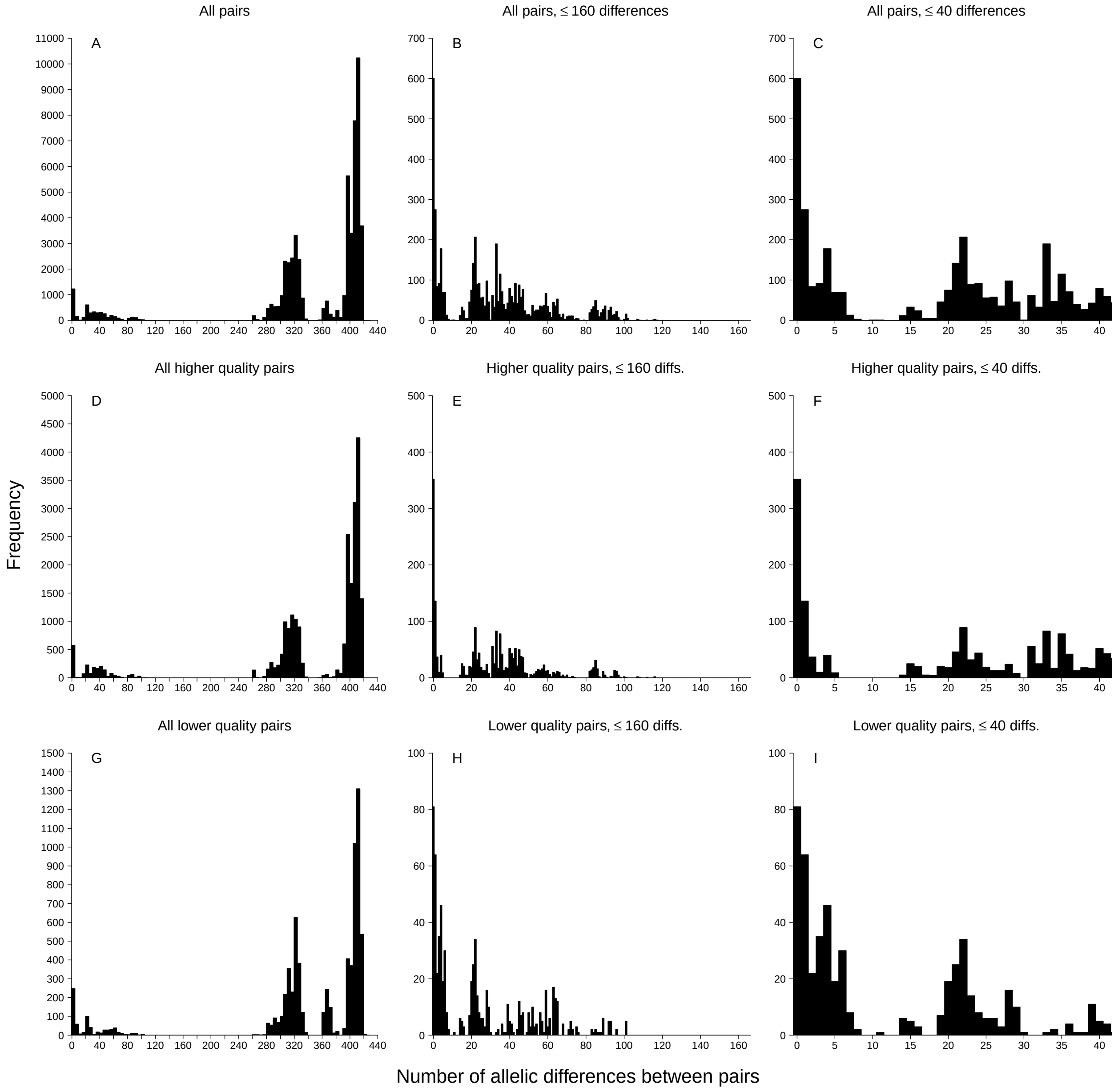


Supplementary figure 1: Histograms of allelic differences between sequence pairs, stratified by sequencing quality

High quality pairs are those where both isolates in the pair had <500 contigs. Lower quality pairs are those where both isolates in the pair had >500 contigs. Counts in the higher and lower quality histograms do not reach the counts in the all pairs histograms as discordant pairs (one higher quality, one lower quality) are also included in the "all pairs" histograms. Note also that y-axis scale differs between each row of histograms.

# Supplementary figure 2

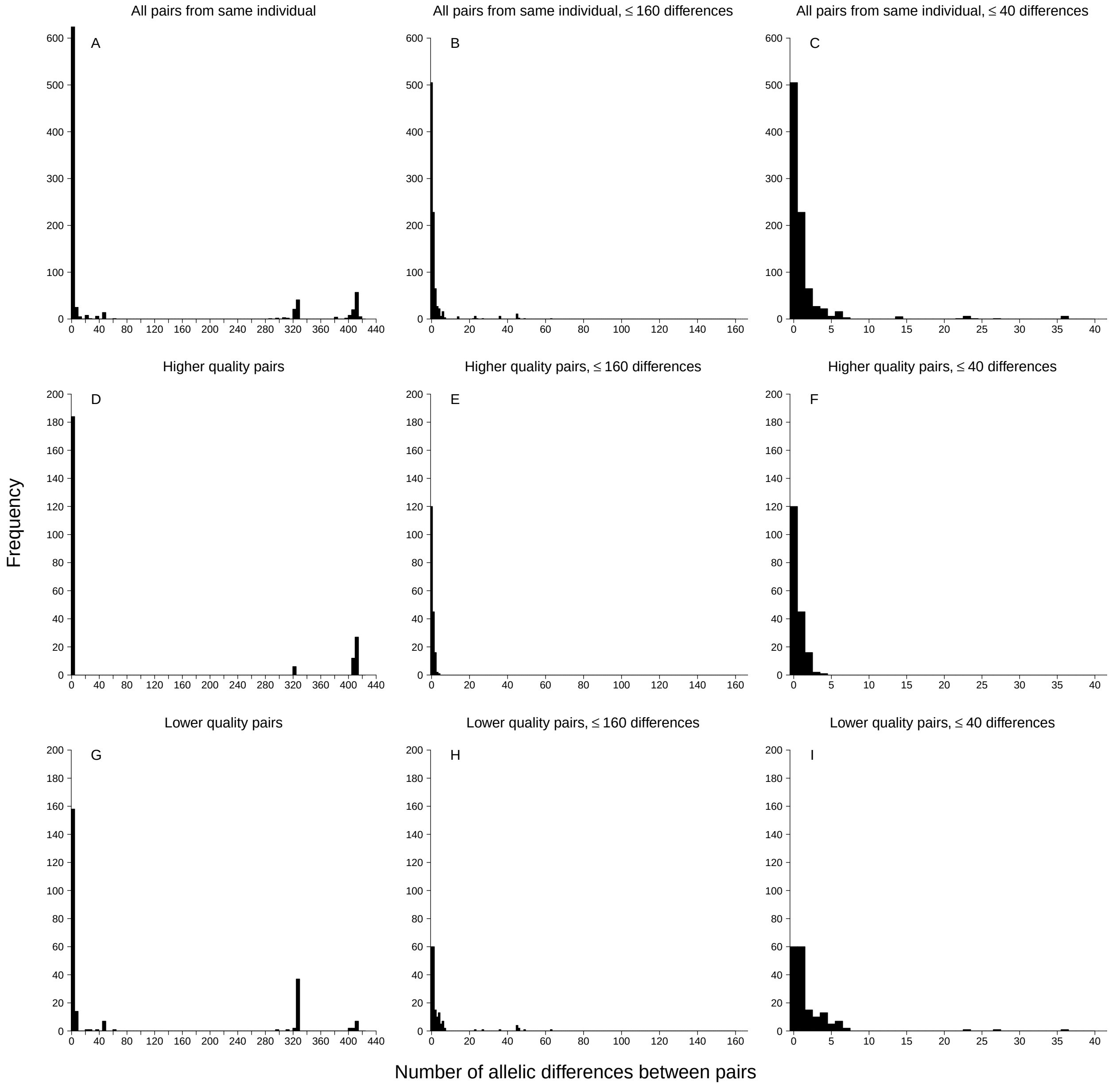


Supplementary figure 2: Histograms of allelic differences between sequence pairs from the same individual, stratified by sequencing quality

High quality pairs are those where both isolates in the pair had <500 contigs. Lower quality pairs are those where both isolates in the pair had >500 contigs. Counts in the higher and lower quality histograms do not reach the counts in the all pairs histograms as discordant pairs (one higher quality, one lower quality) are also included in the "all pairs" histograms. Note also that y-axis scale differs between each row of histograms.
